# First- and third-order chromatic aberrations in Glaser magnetic lens for object magnetic immersion

**DOI:** 10.1016/j.heliyon.2023.e22825

**Published:** 2023-11-24

**Authors:** Aymen Amer, Ahmad K. Ahmad

**Affiliations:** a Al-Quds Power Plant, General Company of Electricity Production/Middle Region, Ministry of Electricity, Baghdad, Iraq; b Department of Laser and Optoelectronics, College of Engineering, Al-Nahrain University, Baghdad, Iraq

**Keywords:** Electron lenses, Differential algebra (DA), Chromatic aberrations, OMI effect, COSY INFINITY 10

## Abstract

In this paper, the Gaussian optical properties and the first- and third-order isotropic and anisotropic chromatic aberrations of the Glaser magnetic lens have been derived analytically and then calculated numerically based on two methods: the differential-algebraic DA and the electron optical aberration integrals. These coefficients have been recalculated for object magnetic immersion OMI. The expressions of chromatic aberration coefficients with the inclusion of the OMI effect of round electron lenses were not published in the literature until the present work. The numerical results of high-order chromatic aberration coefficients of Glaser magnetic lenses calculated using the DA method are shown to be in excellent agreement with those calculated using the integral aberration method and with the minor relative errors of order (10^−7^- 10^−8^), indicating that all the formulas for the chromatic aberration coefficients are entirely correct. For this purpose, COSY INFINITY 10 and Mathematica 11 were used, and both proved to be excellent computer programs for this work.

## 1 Introduction

Many researchers have studied and analyzed the high-order aberrations of different electron optical lens systems using the Differential Algebra DA method. In the years 1992–2001 the authors Zhixiong Liu, Jiye Ximen studied the high-order spherical aberration up to the ninth order and the chromatic aberration up to the third order for electrostatic immersion and Einzel lenses analytically and calculated numerically by means of Mathematica [[1], [2], [3], [4]].

The author Zhixiong Liu has completed his research on the subject of calculating high-order aberrations on round electron lenses across the years 2002–2018 using the differential-algebraic method and the analytical method with the aid of the Mathematica program. He applied the modern map method to the third- and ﬁfth-order aberration analysis of electromagnetic lenses. He also emphasized that there exist three types of differential-algebraic (DA) descriptions for electron optical aberrations. The numerical results of third- and ﬁfth-order aberrations of a given electromagnetic lens computed through the DA technique are shown to be in excellent agreement with those evaluated using electron optical aberration integrals. From his work, one can conclude that the DA technique is concise, efﬁcient, and precise for electron optical aberration analysis and the code COSY INFINITY he used, has been proven to be an excellent computer code for such purposes [[5], [6], [7], [8], [9], [10]].

Liping Wang et al., In 2008, They developed the differential-algebraic (DA) method for the analysis of the aberration of electron mirrors, and a software package MIRROR_DA was developed [11,12].

Min Cheng et al. 2002–2006 applied the diﬀerential algebraic method to analyze and calculate arbitrary order curvilinear-axis combined geometric–chromatic aberrations of electron optical systems. As an example, the Glaser's bell-shaped magnetic electron lens has been studied and the second and third order combined geometric–chromatic aberration coefﬁcients have been calculated [[13], [14], [15], [16]].

Yongfeng Kang et al., in recent years, have used the diﬀerential algebraic method to study and analyze the high-order aberrations (geometrical and chromatic aberrations) up to the fifth order, for different electron optical systems like the multi-offset cylindrical lens, and the Wien filters. They also applied the DA method on the quadrupole, octupole, and practical hexapole correctors whose electric/magnetic fields are in the form of discrete arrays. The correctors include round lenses and hexapole lenses [[17], [18], [19], [20]]. Also, Yongfeng Kang et al., propose a new approach, based on the differential-algebraic (DA) method, for calculating high-order aberration coefficients for variable axis lens (VAL) systems. They trace only one electron trajectory in such a system in lieu of using the straight optical axis as a reference. Then, the linear optical properties and the geometric aberration and chromatic aberration coefficients up to the 5th rank are calculated (with high accuracy) [21].

Tomáš Radlička in 2019 uses the DA method in the optimization of a hexapole corrector of spherical aberration for the transmission regime of the standard scanning electron microscope [22].

The numerical calculation of high-order aberrations for a given electromagnetic lens is carried out using two completely different methods, the DA technique and electron optical aberration integrals to make a cross-check between them. For the analysis of aberrations in electron optical rotationally symmetric systems, there are three different DA [23] descriptions available: the DA description in fixed coordinates, rotating coordinates, and hybrid coordinates [24]. For such systems, the rotating coordinate frame is always used to simplify the analytical expression of the paraxial trajectory equation.

As a result, the aberration is expanded in rotating coordinates and must be corrected by the object magnetic immersion (OMI) effect [7] when the object immerses in the lens magnetic field. It should be noted that Seman [25] was the first to discuss the OMI effect with magnetic lenses. The OMI effect for chromatic aberration coefficients of the magnetic round lens was calculated only for electric perturbation because the results of electric and magnetic perturbations are equal.

The DA description in rotating coordinates becomes very complex for aberrations above the third order. As a result, the general electron trajectory equation and the transfer map expressions must be in rotating coordinates, from which they were previously deduced in Ref. [26].

Two entirely separate methods are used to calculate the first- and third-order aberrations numerically for a particular magnetic Glaser lens. Cross-checking is done between the electron optical aberration integrals and the DA technique. Even when small order errors are present (10^−7^-10^−8^), the results are remarkably stable. It is concluded that COSY INFINITY 10 [27,28] is a great computer code for this work and that the DA descriptions are comprehensive, effective, and exact.

## 2 Theory

### 2.1 First and third-order aberrations of the magnetic lens in electron optics

The chromatic aberration expansion can be divided into two components, the intrinsic $\Delta r_{ci}$ and combined $\Delta r_{cc}$ chromatic aberration [10]. The total expansions ($\Delta r_{c}=\Delta r_{ci}+\Delta r_{cc}$) of the first- and third-order chromatic aberrations of combined electromagnetic lenses under electric perturbation in a vector form are [29,30]:

(1) $$\Delta r_{c1}=-\frac{\Delta V}{V}\left[\left(C_{fc}r_{o}^{\prime }+c_{fc}{r^{\prime }}_{o}^{*}\right)+\left(C_{mc}r_{o}+c_{mc}r_{o}^{*}\right)\right]$$

(2) $$\Delta r_{c3}=-\frac{\Delta V}{V}[\left(B_{c}{r_{o}}^{\prime }+b_{c}{r^{\prime }}_{o}^{*}\right)\left({r_{o}}^{\prime }.{r_{o}}^{\prime }\right)+\left(F_{1c}r_{o}+f_{1c}r_{o}^{*}\right)\left({r_{o}}^{\prime }.{r_{o}}^{\prime }\right)+\left(F_{2c}r_{o}^{\prime }+f_{2c}{r^{\prime }}_{o}^{*}\right)\left({r_{o}}^{\prime }.r_{o}\right)+\left(C_{c}r_{o}+c_{c}r_{o}^{*}\right)\left({r_{o}}^{\prime }.r_{o}\right)+\left(D_{c}{r_{o}}^{\prime }+d_{c}{r^{\prime }}_{o}^{*}\right)\left(r_{o}.r_{o}\right)+\left(E_{c}r_{o}+e_{c}r_{o}^{*}\right)\left(r_{o}.r_{o}\right)]$$

According to Equation (1) there are 2 isotropic (uppercase letters) and 2 anisotropic (lowercase letters) first order aberration coefficients: $C_{fc},c_{fc}$ (Central), $C_{mc},c_{mc}$ (Magnification). According to Equation (2) there are 6 isotropic (uppercase letters) and 6 anisotropic (lowercase letters) third order aberration coefficients: B_c_ (chromatic spherical aberration), $F_{1c},f_{1c}$ (first chromatic coma), $F_{2c},f_{2c}$ (second chromatic coma), $C_{c},c_{c}$ (chromatic astigmatism), $D_{c},d_{c}$ (chromatic field curvature), and $E_{c},e_{c}$ (chromatic distortion) [10,31]. equations (3), (4), (5) show the OMI correction formulas of first- and third-order chromatic aberration coefficients have been derived analytically for the first time by using the Mathematica 11 program [32],

(3) $$\begin{array}{l} C_{fcm}=C_{fc},C_{mcm}=C_{mc}+c_{\text{fc}}\theta_{o}^{\prime }\text{,}\\ c_{fcm}=c_{fc},c_{mcm}=c_{mc}-C_{\text{fc}}\theta_{o}^{\prime }\text{,} \end{array}$$

(4) $$\begin{array}{l} B_{cm}=B_{c},F_{1cm}=F_{1c}+3b_{c}\theta_{o}^{\prime }\text{,}\\ F_{2cm}=F_{2c}-2b_{c}\theta_{o}^{\prime },C_{cm}=C_{c}+2\;f_{1c}\theta_{o}^{\prime }+f_{2c}\;\theta_{o}^{\prime }\;-2B_{c}{\theta^{\prime }}_{o}^{2}\text{,}\\ D_{cm}=D_{c}\;-2\;f_{1c}\theta_{o}^{\prime }+3B_{c}{\theta^{\prime }}_{o}^{2},E_{cm}=E_{c}+d_{c}\;\theta_{o}^{\prime }+F_{1c}{\theta^{\prime }}_{o}^{2}+b_{c}{\theta^{\prime }}_{o}^{3}\text{,} \end{array}$$

(5) $$\begin{array}{l} b_{cm}=b_{c},f_{1cm}=f_{1c}-3B_{c}\theta_{o}^{\prime }\text{,}\\ f_{2cm}=f_{2c}+2B_{c}\theta_{o}^{\prime },c_{cm}=c_{c}-2\;F_{1c}\theta_{o}^{\prime }\;-F_{2c}\;\theta_{o}^{\prime }\;-2B_{c}{\theta^{\prime }}_{o}^{2}\text{,}\\ d_{cm}=d_{c}+2\;F_{1c}\theta_{o}^{\prime }+3b_{c}{\theta^{\prime }}_{o}^{2},e_{cm}=e_{c}\;-D_{c}\;\theta_{o}^{\prime }+f_{1c}{\theta^{\prime }}_{o}^{2}\;-B_{c}{\theta^{\prime }}_{o}^{3}\text{,} \end{array}$$

where, $\theta_{o}^{\prime }=\frac{\eta }{2}\;\frac{B\left(z_{o}\right)}{\sqrt{V\left(z_{o}\right)}}$, $\eta =\sqrt{\frac{e}{2m}}$ , *B*(z_o_) and *V*(z_o_) are the magnetic induction and potential function respectively at the object plane z_o_. The suffix " m" indicates that the aberrations due to the OMI effect.

B. DA description for first and third order chromatic aberrations

The general electron trajectory equation can be followed through a DA integrator from the object plane $z_{o}$ to the image plane $z_{i}$ in fixed coordinates to obtain the DA descriptions in fixed and rotating coordinates. The relationship between chromatic aberration coefficients and the map element was created in Ref. [26]. The first-order isotropic and anisotropic chromatic aberration coefficients with the inclusion of the OMI effect have been found in comparison the DA characterization of rotational coordinates [33] with equations (1), (2) under the electric perturbation:

(6) $$\begin{array}{l} C_{fcm}=\left[M_{c}\left(1,0100\right)\cos\;\vartheta_{i}+M_{c}\left(3,0100\right)\sin\;\vartheta_{i}\right]\;/\left(-\frac{\Delta V}{V}\right)\text{,}\\ C_{mcm}=\left[M_{c}\left(1,1000\right)\cos\;\vartheta_{i}+M_{c}\left(3,1000\right)\sin\;\vartheta_{i}\right]\;/\left(-\frac{\Delta V}{V}\right)\text{,}\\ c_{fcm}=\left[M_{c}\left(3,0100\right)\cos\;\vartheta_{i}-M_{c}\left(1,0100\right)\sin\;\vartheta_{i}\right]\;/\left(-\frac{\Delta V}{V}\right)\text{,}\\ c_{mcm}=\left[M_{c}\left(3,1000\right)\cos\;\vartheta_{i}-M_{c}\left(1,1000\right)\sin\;\vartheta_{i}\right]\;/\left(-\frac{\Delta V}{V}\right)\;\text{;} \end{array}$$

The third-order isotropic chromatic aberration coefficients with OMI effect under electric perturbation are:

(7) $$\begin{array}{l} B_{cm}=\left[M_{c}\left(1,0300\right)\cos\;\vartheta_{i}+M_{c}\left(3,0300\right)\sin\;\vartheta_{i}\right]/\left(-\frac{\Delta V}{V}\right)\text{,}\\ F_{1cm}=\left[M_{c}\left(1,1002\right)\cos\;\vartheta_{i}+M_{c}\left(3,1002\right)\sin\;\vartheta_{i}\right]/\left(-\frac{\Delta V}{V}\right)\text{,}\\ F_{2cm}=\left[M_{c}\left(1,0111\right)\cos\;\vartheta_{i}+M_{c}\left(3,0111\right)\sin\;\vartheta_{i}\right]/\left(-\frac{\Delta V}{V}\right)\text{,}\\ C_{cm}=\left[\left(M_{c}\left(1,1011\right)\cos\;\vartheta_{i}+M_{c}\left(3,1011\right)\sin\;\vartheta_{i}\right)/2\right]/\left(-\frac{\Delta V}{V}\right)\text{,}\\ D_{cm}=\left[M_{c}\left(1,0120\right)\cos\;\vartheta_{i}+M_{c}\left(3,0120\right)\sin\;\vartheta_{i}\right]/\left(-\frac{\Delta V}{V}\right)\text{,}\\ E_{cm}=\left[M_{c}\left(1,3000\right)\cos\;\vartheta_{i}+M_{c}\left(3,3000\right)\sin\;\vartheta_{i}\right]/\left(-\frac{\Delta V}{V}\right)\;\text{;} \end{array}$$

The third order anisotropic chromatic aberration coefficients with OMI effect under electric perturbation are:

(8) $$\begin{array}{l} b_{cm}=\left[M_{c}\left(3,0300\right)\cos\;\vartheta_{i}-M_{c}\left(1,0300\right)\sin\;\vartheta_{i}\right]/\left(-\frac{\Delta V}{V}\right)\text{,}\\ f_{1cm}=\left[M_{c}\left(3,1002\right)\cos\;\vartheta_{i}-M_{c}\left(1,1002\right)\sin\;\vartheta_{i}\right]/\left(-\frac{\Delta V}{V}\right)\text{,}\\ f_{2cm}=\left[M_{c}\left(3,0111\right)\cos\;\vartheta_{i}-M_{c}\left(1,0111\right)\sin\;\vartheta_{i}\right]/\left(-\frac{\Delta V}{V}\right)\text{,}\\ c_{cm}=\left[\left(M_{c}\left(3,1011\right)\cos\;\vartheta_{i}-M_{c}\left(1,1011\right)\sin\;\vartheta_{i}\right)/2\right]/\left(-\frac{\Delta V}{V}\right)\text{,}\\ d_{cm}=\left[M_{c}\left(3,0120\right)\cos\;\vartheta_{i}-M_{c}\left(1,0120\right)\sin\;\vartheta_{i}\right]/\left(-\frac{\Delta V}{V}\right)\text{,}\\ e_{cm}=\left[M_{c}\left(3,3000\right)\cos\;\vartheta_{i}-M_{c}\left(1,3000\right)\sin\;\vartheta_{i}\right]/\left(-\frac{\Delta V}{V}\right)\;\text{;} \end{array}$$

## 3 Results and discussion

The Glaser magnetic lens [31] was chosen, whose magnetic induction distributions has the form as follows;

(9) $$B\left(z\right)=B_{0}/1+{\left(z/d\right)}^{2}\text{,}$$

**Fig. 1:**
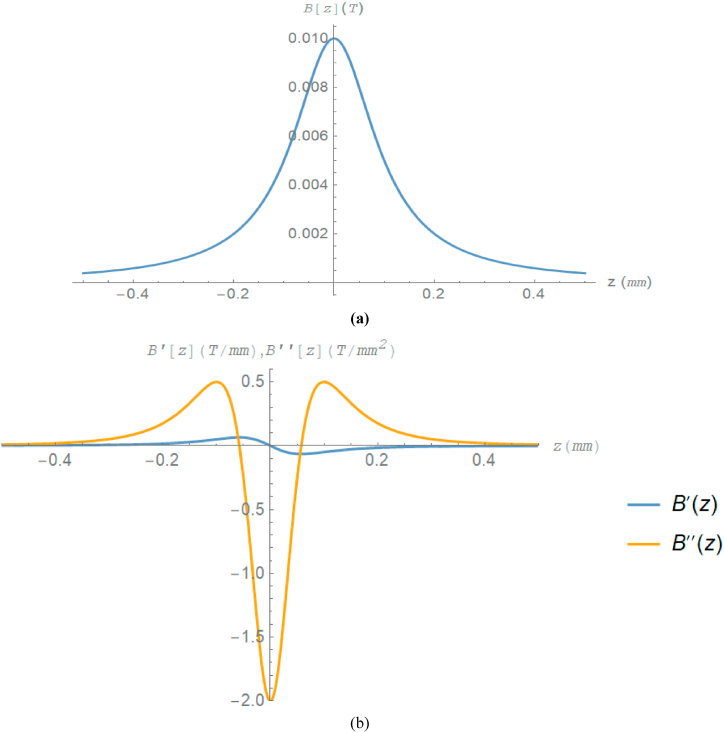
(a) The axial magnetic field distribution *B*(z) (b) Its first and second derivatives along the optical axis z.

*B*_o_ is the highest field in Tesla, and *d* is the field's half-width in millimeters. With the parameters (*B*_o_ = 0.01T, *d* = 0.1 mm) of equation (9) plotted by the Mathematica 11 program, Fig. 1 displays the distribution of the axial field B(z) and its first (dashed line) and second derivative along the optical axis z in the range of $z_{o}=-\;0.3$ mm for the object plane and $z_{i}=0.3$ mm for the image plane for the Glaser magnetic lens. The transfer map in rotating coordinates was generated by an 8th-order Runge-Kutta integrator [34] to track the trajectory equations. Then, for the Glaser magnetic lens, all first- and third-order chromatic aberration coefficients were determined (both with and without taking the OMI effect into account) [5].

Obviously, based on Fig. 1b, the lens has three magnetic poles because the second derivative of the axial magnetic field *B*(z) has two inflection points. The findings of the DA technique and analytical method using the parameters (d = 0.1 mm, *B*_o_ = 0.01 T) of the COSYINFINITY10 program are shown in Table 1 for a Glaser magnetic lens. The results are summarized in this table, with a relative error of order ranging from (10^−5^ to 10^−7^) and a high degree of agreement between the two approaches.

**Table 1:** The Gaussian optical properties for Glaser magnetic lens were calculated by the DA method and analytically with parameters (*d* = 0.1 mm, *B*_*o*_ = 0.01T).

	M	M_s_	F
Analytic Sol.	1	1	−0.132142669002 × 10^5^
DA Method	0.999976227573	1.000023772992142	−0.132142668998 × 10^5^
Relative error	2.377 × 10^−5^	−2.377 × 10^−5^	−4 × 10^−7^

Due to the increasing the values of the field's half-width *d* and the highest magnetic field *B*_*o*_, the magnetic induction distribution *B*(z) and the angle $\theta_{o}^{\prime }$ will also increase, leading to an increasing the isotropic third order chromatic aberration coefficients according to equation (4), as shown in Table 2, Table 3.

**Table 2:** The 3rd order isotropic chromatic aberration coefﬁcients for various half-width (d) under *B*_*o*_ = 0.01 T.

*d* (mm)	B_c_	F_1c_	F_2c_	C_c_	D_c_	E_c_
0.1	−0.30398027	0.28949322 × 10^−4^	0.76055465 × 10^−4^	−0.54073220 × 10^−4^	0.45035722 × 10^−4^	−0.44303350 × 10^−8^
0.2	−0.10777675 × 10^2^	0.25582875 × 10^−2^	0.32771091 × 10^−2^	−0.10839848 × 10^−3^	0.89916707 × 10^−4^	−0.17720025 × 10^−7^
0.3	−0.22742412 × 10^3^	0.10367902 × 10^−1^	0.25308332 × 10^−1^	−0.16335108 × 10^−3^	0.13446737 × 10^−3^	−0.39871535 × 10^−7^
0.4	−0.13453329 × 10^4^	0.12195875 × 10^−1^	0.22610671 × 10^−1^	−0.21683410 × 10^−3^	0.17914498 × 10^−3^	−0.70874626 × 10^−7^

**Table 3:** The 3rd order isotropic chromatic aberration coefﬁcients for various maximum ﬁeld B_o_ under *d* = 1 mm.

*B*_*o*_ (T)	B_c_	F_1c_	F_2c_	C_c_	D_c_	E_c_
0.01	−0.30398027	0.28949322 × 10^−4^	0.76055465 × 10^−4^	−0.54073220 × 10^−4^	0.45035722 × 10^−4^	−0.44303350 × 10^−8^
0.02	−0.11516828 × 10^1^	0.73722536 × 10^−4^	0.15060682 × 10^−2^	−0.21638951 × 10^−3^	0.18002717 × 10^−3^	−0.70913749 × 10^−7^
0.03	−0.23972812 × 10^2^	0.20459367 × 10^−2^	0.81772818 × 10^−2^	−0.48705521 × 10^−3^	0.40496618 × 10^−3^	−0.35897541 × 10^−6^
0.04	−0.27389014 × 10^2^	0.30826887 × 10^−2^	0.17144121 × 10^−1^	−0.86693269 × 10^−3^	0.71952057 × 10^−3^	−0.11345448 × 10^−5^

For Glaser magnetic lenses with and without the OMI effect, the results of the DA and aberration integral approach for the first-order isotropic and anisotropic chromatic aberration coefficients are shown in Table 4 under the same conditions as in Table 1. One can notice that effect of adding the OMI on the first order chromatic aberration coefficients is insignificant, and there is a good consistency in the values calculated by both methods.

**Table 4:** First order isotropic and anisotropic chromatic aberration with and without inclusion OMI effect for Glaser magnetic lens under the same conditions in Table 1.

Aberr. Coeff.	DA Method	Aberr. Integral	Relative errors
*C*_*fc*_	0.27595869 × 10^−5^	0.27595866 × 10^−5^	1.0871192 × 10^−7^
*C*_*mc*_	−0.47619048	−0.47619046	4.2000000 × 10^−8^
*c*_*fc*_	−0.16039188 × 10^−5^	−0.16039186 × 10^−5^	1.2469459 × 10^−7^
*c*_*mc*_	0.32836161 × 10^−2^	0.32836160 × 10^−2^	0.3 × 10^−7^
*C*_*fcm*_	0.27595869 × 10^−5^	0.27595869 × 10^−5^	0.0000000
*C*_*mcm*_	−0.47619048	−0.47619048	0.0000000
*c*_*fcm*_	−0.16039188 × 10^−5^	−0.16039188 × 10^−5^	0.0000000
*c*_*mcm*_	0.32836098 × 10^−2^	0.32836097 × 10^−2^	3.0454288 × 10^−8^

The results of the DA and aberration integral methods for the third-order isotropic and anisotropic chromatic aberration coefficients for a Glaser magnetic lens with and without the OMI effect are shown in Table 5, Table 6 respectively, under the same conditions as in Table 1. From these two tables one can notice that there is a significant effect of adding the OMI on the values of the third order chromatic aberration coefficients, especially for the anisotropic chromatic aberration coefficients values in Table 6, where we notice a reduction in the values of the aberration coefficients.

**Table 5:** Third order isotropic chromatic aberration coefficients with and without inclusion OMI for Glaser magnetic lens under the same conditions used in Table 1.

Aberr. Coeff.	DA Method	Aberr. Integral	Relative Errors
*B*_*c*_	0.30398027	0.30398027	0.0000000
*F*_*1c*_	0.28949322 × 10^−4^	0.28949319 × 10^−4^	1.0362937 × 10^−7^
*F*_*2c*_	0.76055465 × 10^−4^	0.76055461 × 10^−4^	5.2593196 × 10^−8^
*C*_*c*_	−0.54073220 × 10^−4^	−0.54073216 × 10^−4^	7.3973771 × 10^−8^
*D*_*c*_	0.45035722 × 10^−4^	0.45035720 × 10^−4^	4.4409191 × 10^−8^
*E*_*c*_	−0.44303350 × 10^−8^	−0.44303349 × 10^−8^	2.2571650 × 10^−8^
*B*_*cm*_	0.30398027	0.30398027	0.0000000
*F*_*1cm*_	−0.87726704 × 10^−4^	−0.87726701 × 10^−4^	3.4197113 × 10^−8^
*F*_*2cm*_	−0.36870543 × 10^−4^	−0.36870542 × 10^−4^	2.7121922 × 10^−8^
*C*_*cm*_	0.57098477 × 10^−4^	0.57098476 × 10^−4^	1.7513602 × 10^−8^
*D*_*cm*_	−0.49540044 × 10^−4^	−0.49540042 × 10^−4^	4.0371381 × 10^−8^
*E*_*cm*_	0.47956465 × 10^−8^	0.47956463 × 10^−8^	4.1704492 × 10^−8^

**Table 6:** Third order anisotropic chromatic aberration coefficients with and without inclusion OMI for Glaser magnetic lens under the same conditions used in Table 1.

Aberr. Coeff.	DA Method	Aberr. Integral	Relative errors
*b*_*c*_	0.16929457 × 10^−1^	0.16929457 × 10^−1^	0.0000000E+00
*f*_*1c*_	0.24700608 × 10^−2^	0.24700605 × 10^−2^	1.2145450 × 10^−7^
*f*_*2c*_	−0.16273725 × 10^−2^	−0.16273722 × 10^−2^	1.8434624 × 10^−7^
*c*_*c*_	−0.49920581 × 10^−6^	−0.49920578 × 10^−6^	6.0095454 × 10^−8^
*d*_*c*_	0.37160176 × 10^−6^	0.37160174 × 10^−6^	5.3821058 × 10^−8^
*e*_*c*_	0.64270647 × 10^−6^	0.64270645 × 10^−6^	3.1118405 × 10^−8^
*b*_*cm*_	0.16929457 × 10^−1^	0.16929456 × 10^−1^	5.9068640 × 10^−8^
*f*_*1cm*_	0.14146717 × 10^−2^	0.14146715 × 10^−2^	1.4137556 × 10^−7^
*f*_*2cm*_	−0.92377976 × 10^−3^	−0.92377973 × 10^−3^	3.2475273 × 10^−8^
*c*_*cm*_	−0.29914836 × 10^−6^	−0.29914835 × 10^−6^	3.3428229 × 10^−8^
*d*_*cm*_	0.23704712 × 10^−6^	0.23704710 × 10^−6^	8.4371411 × 10^−8^
*e*_*cm*_	0.69764032 × 10^−6^	0.69764029 × 10^−6^	4.3002102 × 10^−8^

The result of the DA and aberration integral methods for the third-order isotropic and anisotropic chromatic aberration coefficients for a Glaser magnetic lens with and without the OMI effect which is published for the first time with the aid of COSYINFINITY10 and Mathematica 11 program shown in Table 4, Table 5, Table 6. These results show the important role of the OMI and the good agreement between the result of the DA and aberration integral methods.

## 4 Conclusion

In the present work, first- and third-order isotropic and anisotropic chromatic aberrations in the Glaser magnetic lens have been re-calculated for object magnetic immersion (OMI) for the first time. The DA method with COSYINFINITY10 was applied for the first time, as was the aberration integral method with the Mathematica 11 program. It is confident that there is a good agreement between these two methods despite the minor errors of order (10^−7^-10^−8^). Finally, it should be emphasized that, based on the DA technique, the map method has the great advantage of conciseness and simplicity without sacrificing accuracy.

## Data availability

The data that has been used is confidential.

## CRediT authorship contribution statement

**Aymen Amer:** Visualization, Methodology, Investigation, Data curation, Conceptualization, Funding acquisition, Software, Writing - original draft, Writing - review & editing. **Ahmad K. Ahmad:** Visualization, Supervision, Project administration, Methodology, Investigation, Conceptualization, Writing - review & editing.

## Declaration of competing interest

The authors declare that they have no known competing financial interests or personal relationships that could have appeared to influence the work reported in this paper.
